# Rediscovery and redescription of the sharpshooter *Kogigonalia incarnata* (Germar, 1821), comb. n. (Hemiptera, Cicadellidae, Cicadellini) from the Atlantic Forest of Brazil, with a key to the species of the genus

**DOI:** 10.3897/zookeys.473.3037

**Published:** 2015-01-20

**Authors:** Gabriel Mejdalani, Rodney R. Cavichioli, Roberta Santos Silva, Victor Quintas

**Affiliations:** 1 Departamento de Entomologia, Museu Nacional, Universidade Federal do Rio de Janeiro, Quinta da Boa Vista, São Cristóvão, 20940-040, Rio de Janeiro, RJ, Brasil; 2 Departamento de Zoologia, Setor de Ciências Biológicas, Universidade Federal do Paraná, Caixa Postal 19020, 81531-980, Curitiba, PR, Brasil

**Keywords:** Cicadellinae, leafhopper, morphology, Neotropics, taxonomy

## Abstract

The Brazilian sharpshooter *Tettigonia incarnata* Germar, 1821 was treated as *incertae sedis* in the most comprehensive and recent monograph of the New World Cicadellini. We have been able to identify male and female specimens of *Tettigonia incarnata* from northeastern and southeastern Brazil using high-resolution images of two syntypes deposited in the Museum für Naturkunde, Universität Humboldt, Berlin. Here we transfer *Tettigonia incarnata* to the genus *Kogigonalia* Young, 1977 and provide a detailed redescription of this species, including information on intraspecific color variation. In addition, we provide an updated key to the species of *Kogigonalia*. This is the first record of the genus from Brazil. *Kogigonalia incarnata* **comb. n.** can be recognized, among other features, by the subgenital plates with a distinct emargination at outer margin, aedeagus with a ventral unpaired process near midlength of shaft, and female sternite VII bearing an elongate strong projection on posterior margin.

## Introduction

Six species were included by Young (1977) in the South American sharpshooter genus *Kogigonalia* Young, 1977 (McKamey 2007, Wilson et al. 2009): *Kogigonalia cajana* Young, 1977 (Peru), *Kogigonalia dietzi* Young, 1977 (Venezuela; type species), *Kogigonalia enola* Young, 1977 (French Guiana), *Kogigonalia resoluta* (Melichar, 1926) (Peru), *Kogigonalia spectabilis* (Melichar, 1932) (Colombia, Peru), and *Kogigonalia zarumoidea* Young, 1977 (Colombia). Young (1977: 82) included *Kogigonalia* in his *Dilobopterus* generic group, a diverse assemblage of 27 genera. Within the *Dilobopterus* group, he considered *Kogigonalia* to be closely related to *Poeciloscarta* Stål, 1869, *Cardioscarta* Melichar, 1932, and *Janastana* Young, 1977. *Kogigonalia* can be distinguished from these three genera, as well as from other Cicadellini, by the following combination of features: crown with anterior margin broadly rounded; thorax with pronotal width greater than transocular width of head, lateral margins of pronotum convergent anteriorly; male pygofer well produced posteriorly, without a dorsal lobe; subgenital plates usually not extending posteriorly as far as pygofer apex; styles usually without a lateral lobe; paraphyses, when present, long-stalked and with a pair of narrowly separated divergent rami; female abdominal sternite VII (known only from *Kogigonalia spectabilis* and *Kogigonalia resoluta*) with a pair of elongate lateral processes or projections.

*Tettigonia incarnata* was described by Germar (1821) based on material from Brazil (“habitat in Brasilia”). In his monograph of the New World Cicadellini, Young (1977: 1105) treated *Tettigonia incarnata* as *incertae sedis* because he was not able to examine specimens of this species. We have been able to identify male and female specimens of *Tettigonia incarnata* from northeastern and southeastern Brazil using high-resolution images of two syntypes (see Wilson et al. 2009) deposited in the Museum für Naturkunde, Universität Humboldt, Berlin. Two additional syntypes reside in the Germar collection in the Ivan Franko National University, Lviv (Shydlovskyy and Holovachov 2005, Holovachov 2008) but were not available for study. The original description of Germar (1821) and the reasonably detailed redescription and color figure of the body provided by Signoret (1853) were also very useful, allowing a precise identification of our specimens. Here we transfer *Tettigonia incarnata* to the genus *Kogigonalia* and provide a detailed redescription of this species, including information on intraspecific color variation. In addition, we provide an updated key to the species of the genus. This is the first record of the genus *Kogigonalia* from Brazil.

## Material and methods

Techniques for preparation of male and female genital structures follow Oman (1949) and Mejdalani (1998), respectively. Dissected genital parts are stored in small vials with glycerin and attached below the specimens, as suggested by Young and Beirne (1958). The descriptive terminology adopted herein follows mainly Young (1977), except for the facial areas of the head (Hamilton 1981, Mejdalani 1993, 1998) and the female genitalia (Nielson 1965, Hill 1970). Use of the term gonoplac (= third ovipositor valvula) and the names of the sculptured areas of the first ovipositor valvulae follow Mejdalani (1998). Photographs of the first and second valvulae were taken with a digital camera attached to an optical microscope. The specimens studied belong to the following institutions: Departamento de Entomologia, Museu Nacional, Universidade Federal do Rio de Janeiro (MNRJ, Rio de Janeiro); Coleção Entomológica Prof. José Alfredo P. Dutra, Departamento de Zoologia, Instituto de Biologia, Universidade Federal do Rio de Janeiro (DZRJ, Rio de Janeiro); Coleção de Entomologia Pe. Jesus S. Moure, Departamento de Zoologia, Setor de Ciências Biológicas, Universidade Federal do Paraná (DZUP, Curitiba); and Museum für Tierkunde (MTD, Dresden).

## Results

### Genus *Kogigonalia* Young, 1977

#### Kogigonalia incarnata

Taxon classificationAnimaliaHemipteraCicadellidae

(Germar, 1821) comb. n.

Figs 1 , 2 , 3a–c

##### Remarks.

*Tettigonia incarnata* Germar, 1821: 69. Catalogued (as *Amblyscarta incarnata*) by Metcalf (1965), McKamey (2007), and Wilson et al. (2009). Redescribed by Blanchard (1840: 190) and Signoret (1853: 684, pl. 22, fig. 11). Four syntypes (two males, two females) from “Bahia” (northeastern Brazil) are deposited in the Museum für Naturkunde, Universität Humboldt, Berlin; we have studied high-resolution images (Fig. 3a–b, dorsal view of the body) of a male and a female syntype (see Wilson et al. 2009). Two additional syntypes are deposited in the Germar collection in the Ivan Franko National University, Lviv (Shydlovskyy and Holovachov 2005: 41, Holovachov 2008).

##### Description.

Length of male 10.4–11.3 mm (n = 3), female 10.8–11.9 mm (n = 3).

**Figure 1.**
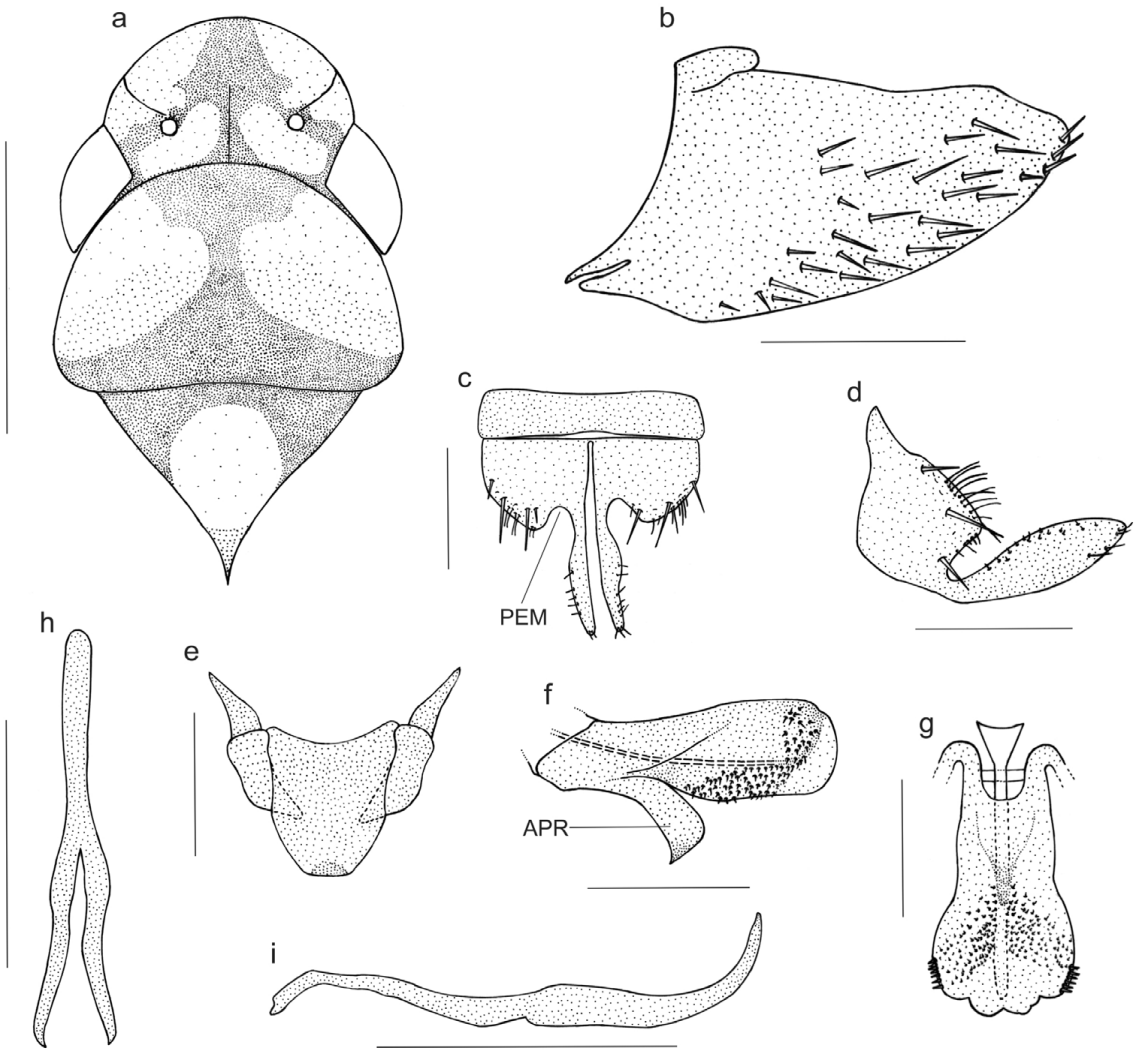
*Kogigonalia incarnata* (Germar, 1821), comb. n. **a** crown, pronotum, and mesonotum, dorsal view. **b–i** male terminalia: **b** pygofer, lateral view **c** valve and subgenital plates, ventral view **d** subgenital plate, lateral view **e** connective and styles, dorsal view **f** aedeagus, lateral view **g** aedeagus, ventral view **h** paraphyses, dorsal view **i** paraphyses, lateral view. APR = aedeagal ventral process; PEM = emargination of subgenital plate. Scale bars: **a** = 2 mm, **b, h, i** = 1 mm, **c–g** = 0.5 mm.

Head (Fig. 1a), in dorsal view, well produced anteriorly, median length of crown approximately 7/10 interocular width and 4/10 transocular width; anterior margin broadly rounded; without carina at transition from crown to face; ocelli located on imaginary line between anterior eye angles, each approximately equidistant between adjacent eye angle and median line of crown; surface without sculpturing or setae; frontogenal sutures extending onto crown and attaining ocelli. Antennal ledges, in dorsal view, not protuberant; in lateral view, with anterior margins oblique and slightly concave. Frons swollen, muscle impressions distinct. Epistomal suture interrupted medially. Clypeus not produced; upper half continuing contour of frons, lower half more nearly horizontal; apex convex.

Thorax (Fig. 1a), in dorsal view, with pronotal width greater than transocular width; pronotum with lateral margins convergent anteriorly; posterior margin rectilinear or slightly concave; disk without sculpturing or setae; dorsopleural carinae declivous anteriorly, incomplete. Mesonotum with scutellum not transversely striate. Forewings coriaceous, venation (except on apical third) not very distinct; membrane well delimited, including first and second apical cells and distal portions of third and fourth apical cells; base of fourth apical cell located more proximally than base of third; with three closed anteapical cells, their bases located more proximally than apex of clavus. Hind wings with vein R_2+3_ incomplete. Hind legs with femoral setal formula 2:1:1; length of first tarsomere greater than combined length of second and third; with two parallel rows of small setae on plantar surface.

Color (Fig. 3a–c). Ground color of anterior dorsum (crown, pronotum, and mesonotum) yellow. Crown with dark brown to black median spear-shaped mark (size variable and may bear lateral extensions, sometimes covering much of coronal surface, with only lateroanterior portions remaining yellow); other variable minor dark brown to black marks also present. Pronotum with conspicuous T-shaped dark brown to black mark, formed by median longitudinal stripe and posterior transverse stripe, anterior pronotal margin with transverse dark brown to black mark at base of “T” (pronotal marks varying from strong to faint or incomplete, sometimes covering much of pronotal surface, with only a pair of lateral areas remaining yellow); lateral portions of disk with variable brown or orange areas. Mesonotum with basal portion largely and variably dark brown to black; posterior portion of scutellum reddish-brown. Ground color of forewings reddish-brown; with or without three large orange or yellow areas, the first and largest on corium and clavus at basal third of wing, the second extending from costal area over clavus and forming transcommissural stripe, and the third extending from costal margin to outer margin of first apical cell (orange or yellow areas, when present, varying from distinct to faint); membrane brown. Face, thorax and legs, and venter of abdomen mostly yellow; frons with or without dark brown to black longitudinal stripe (continued from coronal spear-shaped mark); dorsum of abdomen red; male pygofer reddish.

Male genitalia with pygofer (Fig. 1b), in lateral view, strongly produced posteriorly; posterior margin narrowly rounded; without processes; macrosetae distributed mostly on posterior half and extending anteriorly along ventral margin. Valve (Fig. 1c), in ventral view, subrectangular. Subgenital plates (Fig. 1c–d) much shorter than pygofer; in ventral view, with basal half broad and apical half abruptly and strongly narrowed; transition from broad to narrow portion emarginated; basal half with uniseriate macrosetae; plate surface with scattered microsetae; plates separate from each other throughout their length. Styles (Fig. 1e), in dorsal view, with apophysis short, not extending as far posteriorly as apex of connective, narrowing gradually toward apex, without preapical lobe, with few preapical setae on outer margin. Connective (Fig. 1e), in dorsal view, a large trapezoidal plate; without median keel. Aedeagus (Fig. 1f–g) symmetrical; shaft, in ventral view, expanded apically; in lateral view, with strong, median ventral process on basal half; shaft apex with pair of membranous lobes; shaft surface with pair of areas covered by small spines, extending from median ventral process to lateroapical area, where spines are larger than more basal ones. Paraphyses (Fig. 1h–i), in dorsal view, with both stalk and rami elongate, the former articulated with connective, the latter with apical half curved dorsally.

**Figure 2.**
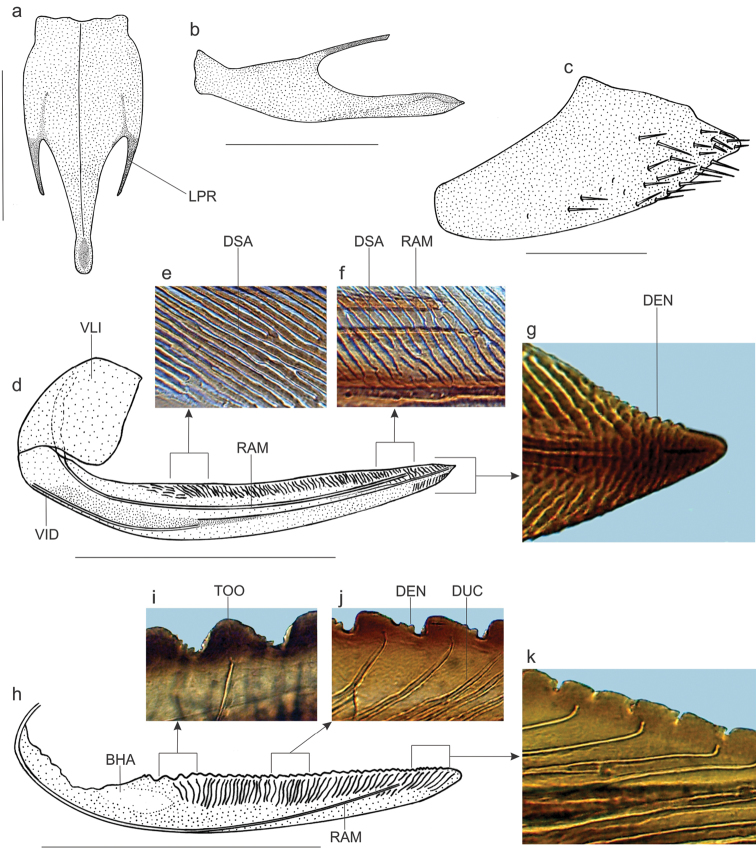
*Kogigonalia incarnata* (Germar, 1821), comb. n., female terminalia: **a** sternite VII, ventral view **b** sternite VII, lateral view **c** pygofer, lateral view **d** first valvifer and valvula, lateral view **e** dorsal sculptured area at basal portion **f** dorsal sculptured area at apical portion **g** apex **h** second valvula, lateral view **i** teeth at basal portion **j** teeth at median portion **k** teeth at apical portion. BHA = basal hyaline area; DEN = denticle; DSA = dorsal sculptured area; DUC = duct; LPR = lateral process of sternite VII; RAM = ramus; TOO = tooth; VID = ventral interlocking device; VLI = first valvifer. Scale bars: **a, b, d, h** = 2 mm, **c** = 1 mm.

Females with abdominal sternite VII (Fig. 2a–b), in ventral view, strongly produced posteriorly; posterior margin with elongate, median strong projection and pair of elongate, but shorter than median projection, lateral spiniform processes; median projection with slight preapical constriction; ventral surface of sternite VII with distinct median longitudinal carina. Internal sternite VIII, in dorsal view, without distinct median or lateral sclerites. First valvifers (Fig. 2d), in lateral view, with anterior and dorsal margins rounded, ventral margin emarginated, posterior margin truncate. Pygofer (Fig. 2c), in lateral view, strongly produced posteriorly; apex narrowly rounded; ventral margin slightly emarginated preapically; macrosetae distributed mostly on posterior portion and extending anteriorly along ventral margin. First valvulae, in ventral view, with basal portion expanded, without processes or projections; in lateral view (Fig. 2d), with apex acute; dorsal margin with approximately 10 preapical denticles (Fig. 2g); dorsal sculptured area (Fig. 2e–f) extended from basal portion to apex of blade, formed mostly by oblique linear processes; ventral sculptured area restricted to apical portion of blade, formed mostly by scale-like processes; ventral interlocking device (Fig. 2d) restricted to basal half of blade, its apical third curved dorsally. Second valvulae (Fig. 2h), in lateral view, slightly expanded beyond basal curvature; basal hyaline area distinct; dorsal margin approximately rectilinear, with about 40 continuous teeth (Fig. 2i–k) that are progressively smaller toward apex; most teeth subtriangular but posterior ones quadrate; few irregular denticles on posterior portion of larger teeth and on ventroapical portion of blade; ventral blade margin convex; without preapical prominence; apex obtuse. Gonoplacs, in lateral view, with basal half narrow and apical half distinctly expanded; apex obtuse; blade with many minute spiniform processes and few macrosetae on apical portion and extending anteriorly along ventral margin.

**Figure 3.**
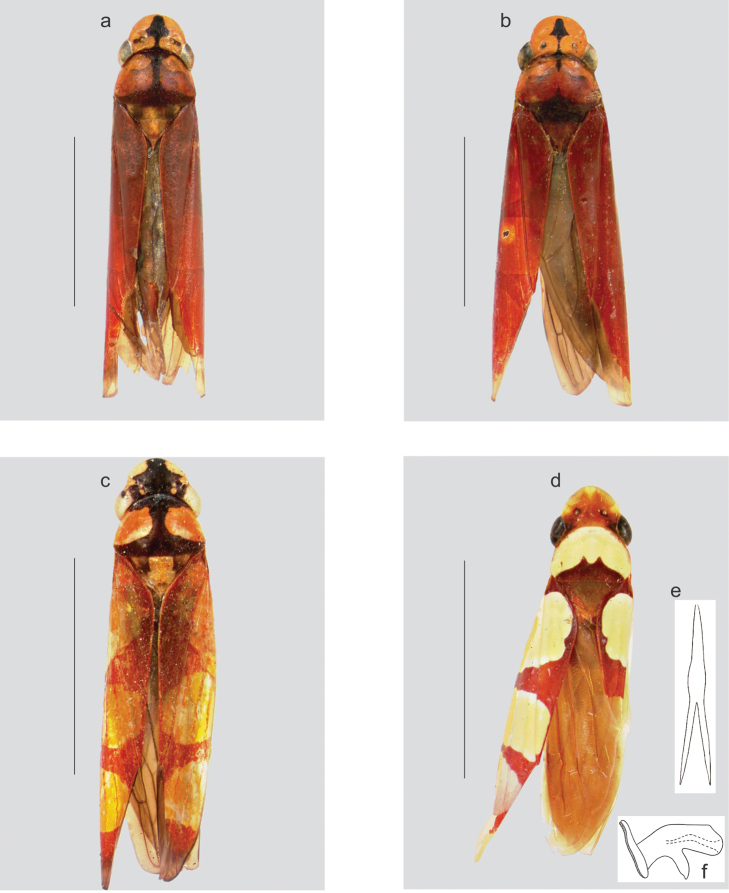
**a–c** color variation in *Kogigonalia incarnata* (Germar, 1821), comb. n., body, dorsal view: **a–b** male and female syntypes, respectively, from the state of Bahia, northeastern Brazil (Museum für Naturkunde, Universität Humboldt, Berlin) **c** female from Brazil **d–f** *Kogigonalia enola* Young, 1977, male holotype from French Guiana (United States National Museum, Washington, D.C.): **d** body, dorsal view **e** paraphyses, dorsal view **f** aedeagus, lateral view. **a–d** reproduced, with permission, from Wilson et al. (2009) **e–f** redrawn from Young (1977). Scale bars = 5 mm.

##### Material examined.

northeastern Brazil: *state of Bahia*: one female (MTD). southeastern Brazil: *state of Espírito Santo*: one male, Santa Teresa, 675 m, 1–2/IV/1969, Exp. Dep. Zool. col. (DZUP); Baixo Guandu, 17/IX/1966, C. Elias col. (DZUP); *state of Rio de Janeiro*: two males and one female, Casimiro de Abreu, Reserva Biológica União, 28–31/I/2013 (one male), 12/XII/2013–27/I/2014 (one male, one female), Lab. Diptera MN[RJ] col., Malaise trap (MNRJ); one male, Silva Jardim, III/1974, F. M. Oliveira col. (DZUP); one male, Magé, 3/III/1978, J. L. Nessimian col. (DZRJ). Brazil: one female, D. Swainson col. (DZUP); one specimen without abdomen (MTD).

### Key to males of *Kogigonalia* and female of *Kogigonalia resoluta* (adapted from Young 1977)

Note: in addition to the present paper, the reader is referred to Young (1977, Figs 169–174) and Wilson et al. (2009) for illustrations and photographs of the external morphology and genital structures of *Kogigonalia* species that will be useful for evaluating the identifications obtained using our key.

**Table:** 

1a	Dorsum red with a pair of yellow maculae on lateroposterior portions of crown and a pair of small yellow marks on lateral margins of pronotum	***Kogigonalia resoluta* (Melichar, 1926)** (known only from female)
1b	Dorsum not as above	**2**
2a	Aedeagus with a large, ventral unpaired process near midlength of shaft and no additional processes (Fig. 1f)	**3**
2b	Aedeagus without such a process or with additional processes	**4**
3a	Subgenital plates, in lateral view, extending approximately as far posteriorly as pygofer apex and, in ventral view, without outer emargination at transition from broad basal portion to narrow apical portion	***Kogigonalia enola* Young, 1977**
3b	Subgenital plates, in lateral view, very short, not extending as far posteriorly as pygofer apex and, in ventral view, with distinct outer emargination at transition from broad basal portion to narrow apical portion (Fig. 1c)	***Kogigonalia incarnata* (Germar, 1821), comb. n.**
4a	Face with at least some black marking	**5**
4b	Face without black marking	**6**
5a	Genae yellow	***Kogigonalia spectabilis* (Melichar, 1932)**
5b	Genae black	***Kogigonalia zarumoidea* Young, 1977**
6a	Pygofer without processes; paraphyses present	***Kogigonalia dietzi* Young, 1977**
6b	Pygofer with a process arising at middle of posterior margin; paraphyses absent	***Kogigonalia cajana* Young, 1977**

## Discussion

The aedeagus and paraphyses of *Kogigonalia incarnata* are very similar to those of *Kogigonalia enola*, a species described by Young (1977) from French Guiana. In these species, the aedeagus bears a large, ventral unpaired process near the midlength of shaft (Figs 1f, 3f) and the paraphyses have both the stalk and rami elongate (Figs 1h–i, 3e). Our assignment of *Tettigonia incarnata* to *Kogigonalia* is based mostly on these remarkable similarities. In addition, the color pattern of the forewings of *Kogigonalia enola* (Fig. 3d) is very similar to that of *Kogigonalia incarnata* (Fig. 3c) specimens that have three large orange or yellow areas on each wing. However, *Kogigonalia incarnata* shows a great deal of intraspecific color variation; the orange or yellow forewing areas vary from distinct to faint or even absent (Fig. 3a–c); the dark marks of crown and pronotum are also variable, even between the syntype specimens from the state of Bahia, northeastern Brazil (Fig. 3a–b). In spite of this color variation, we believe that all specimens herein examined belong in *Kogigonalia incarnata* because all males have the same genitalia morphology (Fig. 1b–i) and some of them match perfectly the color pattern of the syntypes. Likewise, females with distinct color patterns show the same terminalia morphology, including the strongly produced sternite VII (Fig. 2a–b). Similar cases of intraspecific color variation are known in other Cicadellini [e.g., *Macugonalia leucomelas* (Walker, 1851), *Tettisama quinquemaculata* (Germar, 1821), *Versigonalia ruficauda* (Walker, 1851)] and in Proconiini [e.g., *Pseudometopia amblardii* (Signoret, 1855), *Raphirhinus phosphoreus* (Linnaeus, 1758), *Teletusa limpida* (Signoret, 1855)].

## Supplementary Material

XML Treatment for Kogigonalia incarnata
